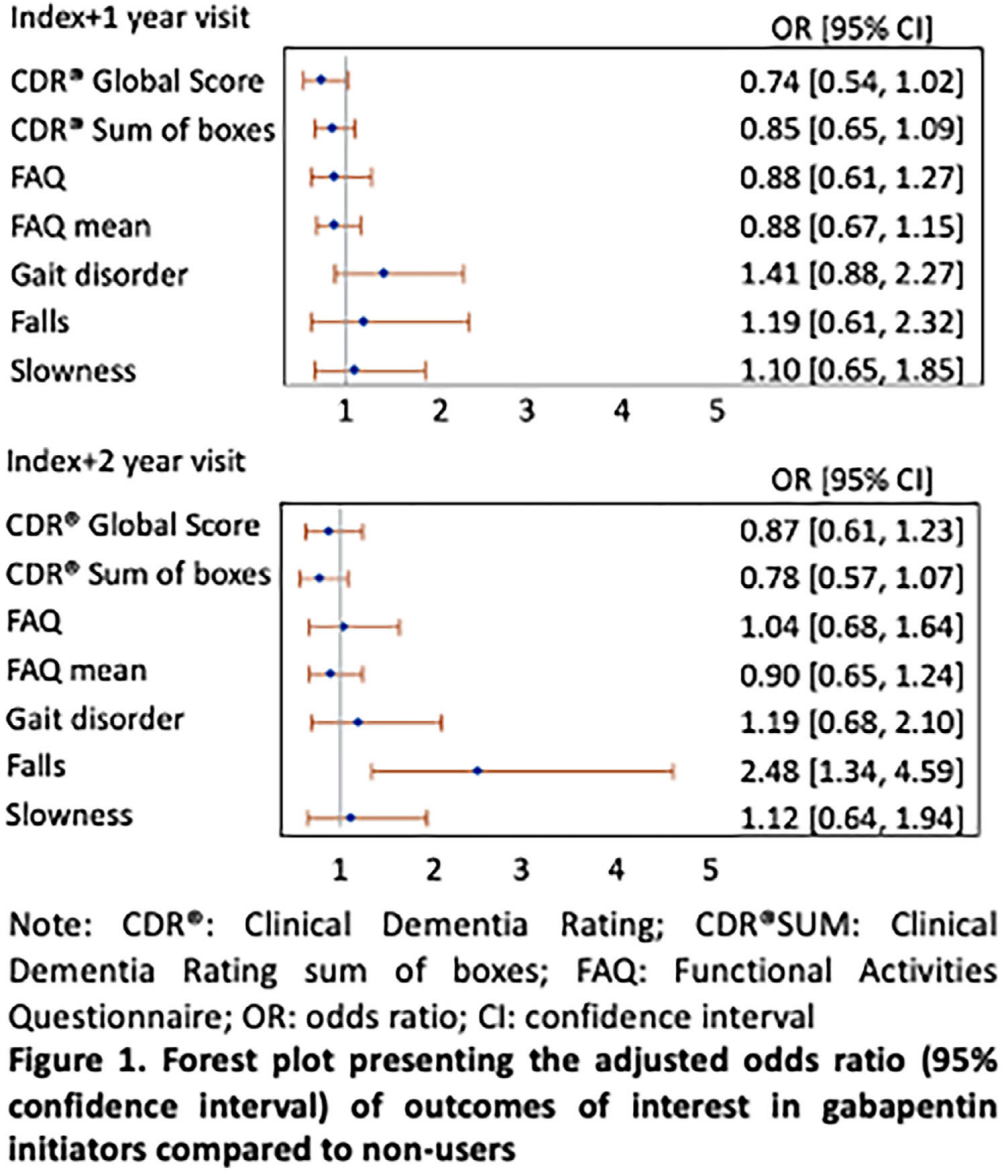# Gabapentin initiation and neurocognitive changes in older adults with cognitive impairment

**DOI:** 10.1002/alz.090204

**Published:** 2025-01-09

**Authors:** GYeon Oh, Daniela C Moga, David W. Fardo, Jordan P. Harp, Erin L. Abner

**Affiliations:** ^1^ University of Kentucky College of Public Health, Lexington, KY USA; ^2^ University of Kentucky / Sanders‐Brown Center on Aging, Lexington, KY USA; ^3^ University of Kentucky College of Pharmacy, Lexington, KY USA; ^4^ Sanders‐Brown Center on Aging, University of Kentucky, Lexington, KY USA

## Abstract

**Background:**

Gabapentin has been increasingly prescribed to older adults for off‐label indications, and accumulating evidence suggests potential for gabapentin misuse and related adverse events. However, the relation between gabapentin initiation and longer‐term neurocognitive changes is not well understood.

**Method:**

A retrospective cohort study was conducted using the National Alzheimer’s Coordinating Center Uniform Data Set (2005‐March 2023). Participants with cognitive impairment at the visit of gabapentin initiation (i.e., index visit) were included. Using the incidence density sampling method, up to 9 non‐users were randomly selected for each initiator. Cognitive decline over one year was defined as any increase in Clinical Dementia Rating global score (CDR®GLOB) or a 1‐point increase in CDR® sum of boxes (CDR®SB). Functional status decline over one year was defined as at least a 3‐point increase in the Functional Activities Questionnaire (FAQ) sum or a 0.3‐point increase of mean of FAQ. Motoric decline over one year was defined as new clinician reports of gait disorder, falls, and slowness. To mitigate confounding and selection bias, joint stabilized inverse probability of treatment weights and censoring weights were used. Generalized estimating equations with an exchangeable working correlation structure were used to fit logistic regression models and account for the weighting, The confounders were selected using directed acyclic graphs. All analyses were conducted comparing index to index+1 and index+2 visits.

**Result:**

For the study of cognitive and functional status decline, we included 505 initiators (mean age [SD]: 78.8 [7.4]; male = 45%) and 4,545 non‐users (79.2 [7.6]; 50.1%). For the study of motor decline, we included 353 initiators (78.3 [7.2]; 42.8%) and 3,177 non‐users (78.5 [7.4]; 48.1%). Gabapentin initiation was not statistically associated with decline on CDR®GLOB, CDR®SB, FAQ sum, or mean FAQ at the index+1 or index+2 visits. However, gabapentin initiation was significantly associated with increased odds of new falls at the index+2 visit (odds ratio [95% confidence interval]: 2.1 [1.1, 3.9]) (Figure 1).

**Conclusion:**

Over 1 or 2 years of follow‐up, gabapentin initiation was not associated with decline in cognitive or functional status but was associated with increased odds of falling among research participants with cognitive impairment.